# Differential structural remodelling of heparan sulfate by chemokines: the role of chemokine oligomerization

**DOI:** 10.1098/rsob.160286

**Published:** 2017-01-25

**Authors:** Douglas P. Dyer, Elisa Migliorini, Catherina L. Salanga, Dhruv Thakar, Tracy M. Handel, Ralf P. Richter

**Affiliations:** 1 Skaggs School of Pharmacy and Pharmaceutical Sciences, University of California, San Diego, La Jolla, CA 92093-0684, USA; 2 Institute of Infection, Immunity and Inflammation, College of Medical, Veterinary and Life Sciences, University of Glasgow, Glasgow G12 8TA, UK; 3 CIC biomaGUNE, 20009 Donostia-San Sebastian, Spain; 4 Département de Chimie Moléculaire, Université Grenoble Alpes-CNRS, 38041 Grenoble Cedex 9, France; 5 School of Biomedical Sciences and School of Physics and Astronomy, University of Leeds, Leeds LS2 9JT, UK

**Keywords:** chemokine, glycosaminoglycan, heparan sulfate, extracellular matrix, glycocalyx, quartz crystal microbalance with dissipation monitoring

## Abstract

Chemokines control the migration of cells in normal physiological processes and in the context of disease such as inflammation, autoimmunity and cancer. Two major interactions are involved: (i) binding of chemokines to chemokine receptors, which activates the cellular machinery required for movement; and (ii) binding of chemokines to glycosaminoglycans (GAGs), which facilitates the organization of chemokines into haptotactic gradients that direct cell movement. Chemokines can bind and activate their receptors as monomers; however, the ability to oligomerize is critical for the function of many chemokines *in vivo*. Chemokine oligomerization is thought to enhance their affinity for GAGs, and here we show that it significantly affects the ability of chemokines to accumulate on and be retained by heparan sulfate (HS). We also demonstrate that several chemokines differentially rigidify and cross-link HS, thereby affecting HS rigidity and mobility, and that HS cross-linking is significantly enhanced by chemokine oligomerization. These findings suggest that chemokine–GAG interactions may play more diverse biological roles than the traditional paradigms of physical immobilization and establishment of chemokine gradients; we hypothesize that they may promote receptor-independent events such as physical re-organization of the endothelial glycocalyx and extracellular matrix, as well as signalling through proteoglycans to facilitate leukocyte adhesion and transmigration.

## 1. Introduction

Glycosaminoglycans (GAGs) are long chains of repeating saccharide units that get attached to protein cores to form proteoglycans that are either inserted into cell membranes or secreted/shed into the extracellular matrix (ECM) [1,2]. For example, heparan sulfate (HS) is a ubiquitous GAG found on almost all cell surfaces where it is attached as part of the HS proteoglycans syndecan and glypican to the membrane and forms a large component of the endothelial glycocalyx [1–3]. Along with the ECM, one function of HS and other GAGs in the glycocalyx is to provide structural support for the physical deposition of a wide number of growth factors, cytokines, chemokines and other ECM proteins [4]. However, more recently this layer has been described as playing a dynamic physical role in controlling the permeability of the endothelium to leukocytes by regulating leukocyte adhesion to the endothelial cells prior to their transmigration through the endothelial cell layer [5–8]. It also physically responds to forces such as shear stress, thereby transducing mechanical signals into cellular responses [9] and undergoes physical remodelling as a consequence of disease [10–13]. The control mechanisms involved in physical regulation of this barrier have yet to be fully described but are thought to be integral factors in inflammation and cell migration [11,14,15], suggesting the potential involvement of chemokines.

Chemokines are a family of chemotactic cytokines that play key roles in mediating leukocyte adhesion and cell migration [16–18]. In addition to binding and activating chemokine receptors on the migrating cells, chemokines bind to GAGs, an interaction that has been demonstrated to be vital for their function *in vivo* [19]. This functional requirement has been demonstrated with GAG-binding-deficient mutants of several chemokines (e.g. CXCL8, CXCL12, CCL2, CCL5 and CCL7) using *in vivo* mouse models of inflammation where the mutants block the function of wild-type (WT) chemokines and/or fail to recruit immune cells [20–26]. The importance of chemokine–GAG interactions has been attributed to the need to localize chemokines to inflammatory environments where they are produced [27], particularly in the presence of convective transport by flow in blood vessels and capillaries. These interactions are also thought to be important for the formation of haptotactic chemokine gradients that provide directional cues for migrating cells [27,28]. However, other mechanisms related to modifying the organization of GAGs on cell surfaces and the ECM may be operative [29]. Along these lines, recent studies demonstrated that the tumour necrosis factor-stimulated gene-6 (TSG-6) can interconnect individual chains of hyaluronan (HA), and thus non-covalently cross-links this GAG [30]. The functional consequence of this cross-linking was suggested to be ‘HA-remodelling’ for regulating leukocyte adhesion and enhancing the sequestration of additional ECM proinflammatory mediators [31]. Similarly, cross-linking of HS by growth factors such as FGF-2 as well as the chemokines CXCL12α and γ has also been demonstrated [29]. These studies involved the use of biophysical techniques referred to as quartz crystal microbalance with dissipation monitoring (QCM-D) and fluorescence recovery after photobleaching (FRAP) to report on physical properties (rigidification and mobility, respectively) of GAG films upon protein binding. The observation that CXCL12α cross-links HS chains by QCM-D and FRAP is in agreement with results from surface plasmon resonance (SPR), which revealed a dependence of chemokine–GAG affinities on the density of the immobilized GAG chains, in a manner suggestive of cross-linking [32,33].

**Figure 1.**
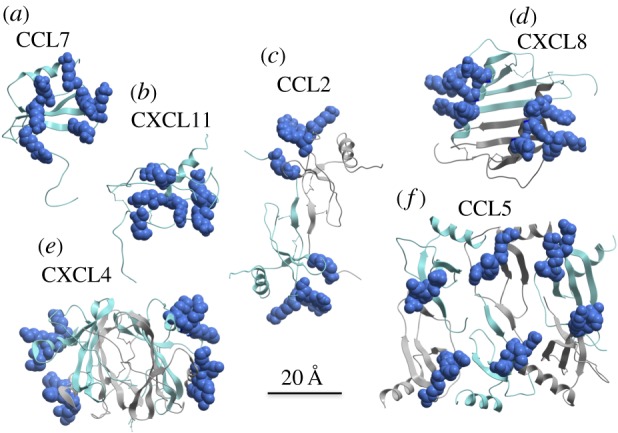
Structures of chemokines used in this study with major GAG-binding residues highlighted. Different subunits of chemokines are shown as grey and cyan ribbons. Highlighted residues (space-filling representation, blue) are those previously identified as being important for binding to GAGs. (*a*) CCL7 (PDB ID 1BO0 [34]; residues highlighted include R14, K18, K19, K22, R24, K46, K49 [32]). (*b*) CXCL11 (PDB ID 1RJT [35]; residues highlighted include K46, K50, K52, K57, K59, R62 [36]). (*c*) CCL2 (PDB ID 1DOM [37]; residues highlighted include R18, K19, R24, K49 [38]). (*d*) CXCL8 (PDB ID 1IL8 [39]; residues highlighted include H18, K20, R60, K64, K67, R68 [40]). (*e*) CXCL4 (PDB ID 1RHP [41]; residues highlighted include K61, K62, K65, K66 [42]). (*f*) CCL5 (PDB ID 5CMD [43], only hexamer shown; residues highlighted include R44, K45, R47 [44]).

The purpose of the present study was to determine whether cross-linking of GAG chains is a common feature of chemokine–GAG interactions, and to provide insight into the underlying structural mechanisms. In particular, prior studies demonstrating that HA induces oligomerization of TSG-6, and that the TSG-6 oligomers act as cross-linkers of HA films [30], motivated us to consider analogous mechanisms with chemokines. Indeed, all chemokines have the same basic tertiary fold (figure 1*a,b*; CCL7 and CXCL11, respectively), and they generally bind and fully activate their receptors as monomers [20,45–47]. However, many chemokines oligomerize, which might facilitate bridging of individual GAG chains as observed for TSG-6 oligomers. Chemokines from the CC subfamily generally form ‘CC-like’ dimers through association of N-termini (figure 1*c*, CCL2) while CXC chemokines generally form ‘CXC-like’ dimers through association of β-sheets [39] (figure 1*d*, CXCL8). Others form larger oligomers, such as tetramers in the case of CXCL4 [41] (figure 1*e*) and polymers in the case of CCL5 [43] (figure 1*f*). As for HA-induced oligomerization of TSG-6 [30], GAGs can also stabilize or induce chemokine oligomerization [32,38]. Thus within the chemokine family, there is a broad range of GAG affinities and oligomerization propensities, as well as effects of GAGs on chemokine oligomerization, that could contribute to chemokine specific effects in modulating the biomechanical and structural properties of endothelial and ECM GAGs.

**Figure 2.**
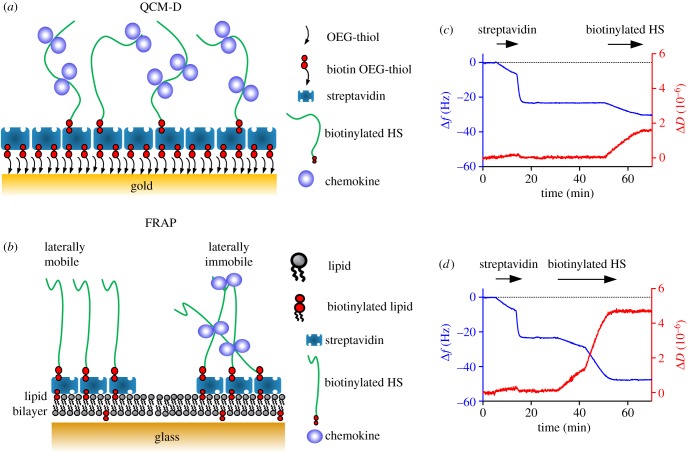
Formation of HS films on a passivated QCM-D sensor and for FRAP. Schematic of surfaces used for (*a*) QCM-D and (*b*) FRAP adapted from [29]. Formation of (*c*) low- and (*d*) high-density HS surfaces on a QCM-D sensor; streptavidin (1 or 20 µg ml^−1^) was flowed over a gold-coated sensor covered with a passivation layer presenting biotin groups until saturation was reached (−24 Hz, no change in dissipation). Biotinylated HS (2 or 5 µg ml^−1^) was then grafted onto this surface to create a low-density HS film (−7 Hz, 1.5 dissipation units) (*c*) or a high-density HS film (−24 Hz, 4.5 dissipation units) (*d*), which could then be used to monitor interactions of HS with chemokines or mutants.

In this study, we characterized six chemokines that have a wide range of GAG-binding affinities and oligomerization propensities, from monomers to polymers (figure 1), for their ability to bind to and modify HS films using QCM-D (figure 2*a*) and FRAP (figure 2*b*). We also examined oligomerization-deficient chemokines to directly probe the role of oligomerization. Our results suggest that chemokines differentially accumulate on and rigidify HS films. In the case of CCL2, CCL5 and CXCL4, we also show that HS chain modification is dependent on their ability to oligomerize and that oligomerization allows chemokines to cross-link HS chains over greater distances than non-oligomerizing counterparts. These results provide insight into the potential of chemokines to modify the physical properties of HS chains in the ECM and the glycocalyx, which may reflect an important aspect of chemokine function—modulating the ECM and endothelial cell barrier function to facilitate leukocyte adhesion and transmigration.

## 2. Results

### 2.1. Chemokines differentially bind to and rigidify heparan sulfate films

In previous studies, we investigated the interaction of several chemokines and oligomerization-deficient chemokine mutants with HS using SPR. In addition to determining interaction affinities and kinetic on/off rates as well as insight into the relative propensity of the chemokines and their mutants to oligomerize on HS, we observed that for some chemokines, the affinities were dependent on the density of the immobilized HS chains [32,33]. These observations led us to hypothesize that some chemokines, particularly those that oligomerize, might cross-link HS chains. In order to test this hypothesis, we set out to determine if the same set of chemokines could modify HS surfaces, possibly through cross-linking, using QCM-D experiments. QCM-D has been previously used to investigate cross-linking/remodelling of HA by TSG-6 [30,48] and, more recently, HS by growth factors FGF-2 and -9, the cytokine IFNγ and chemokines CXCL12α and CXCL12γ [29,49].

In the QCM-D approach, one creates an artificial biomimetic surface by passivating gold-coated QCM-D sensors with a monolayer of oligo ethylene glycol (OEG) thiols doped with biotinylated OEG thiol. The surfaces are then coated with a monolayer of streptavidin, followed by a monolayer of biotinylated HS. The resulting film presents a monolayer of HS whose density can be controlled and interactions with various GAG-binding proteins interrogated (figure 2*a*). Note that in these experiments, HS is biotinylated and then immobilized on streptavidin through its reducing end to mimic attachment to proteoglycans and thereby minimize artificial perturbations of the protein–HS interactions under study.

QCM-D measures two parameters: (i) the frequency, or frequency shift relative to a control (Δ*f*, in Hertz), which is sensitive to changes in areal mass density, and therefore reports on molecular binding events (e.g. binding of HS, streptavidin or chemokine in our study); and (ii) the dissipation, or dissipation shift relative to a control (Δ*D*, in dissipation units, 10^−6^), which reflects changes in the morphology of the biomolecular film on the sensor surface (e.g. relative softness or rigidification). Figure 2*c,d* illustrates the formation of a lower-density (−7 Hz, +1.5 dissipation units) HS surface and a saturated (−24 Hz, +4.5 dissipation units) HS surface, respectively, as previously described [29,49]. The resulting HS films are soft and hydrated, as indicated by the increase in dissipation upon HS addition [50]. The frequency shifts upon HS binding correspond to areal HS densities of 10 and 36 ng cm^−2^, respectively [51]. Changes in frequency and dissipation upon flowing chemokine over the surface can then be used to assess binding to and rigidification of the HS film [49]. The frequency and dissipation corresponding to bound HS and HS film softness, respectively, are set to zero in subsequent figures (described below) to focus exclusively on the effects of chemokine addition.

**Figure 3.**
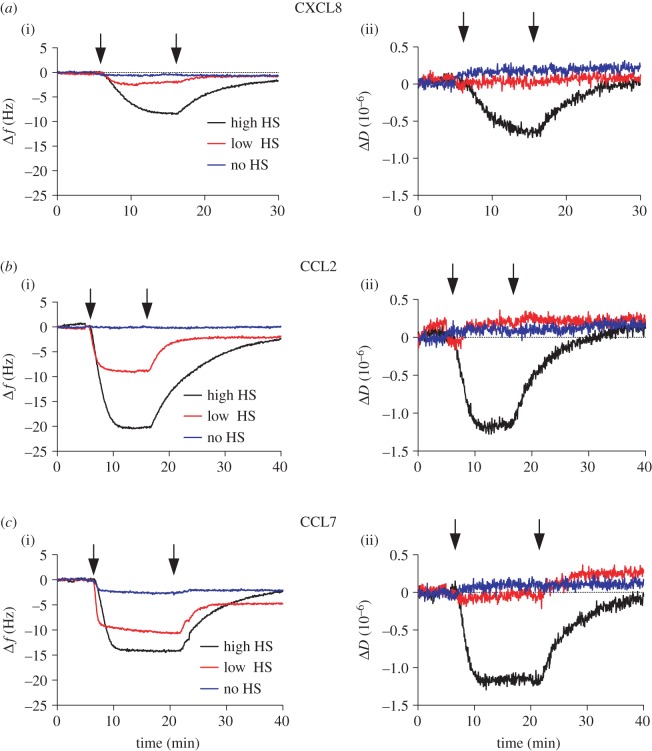
CXCL8-, CCL2- and CCL7-mediated modification of high- and low-density HS films. (*a*) CXCL8, (*b*) CCL2 or (*c*) CCL7 (500 nM) were passed over a QCM-D sensor displaying high-density (high HS) or low-density (low HS) HS films. Alternatively, chemokine was passed over a streptavidin-coated surface with no immobilized HS (no HS) to check non-specific binding (passivation). Subsequent changes in the (i) frequency (a decrease indicates bound chemokine) or (ii) dissipation (a decrease indicates HS film rigidification) as a function of time are plotted. Chemokine injection start and endpoints are indicated by arrows on each curve.

**Figure 4.**
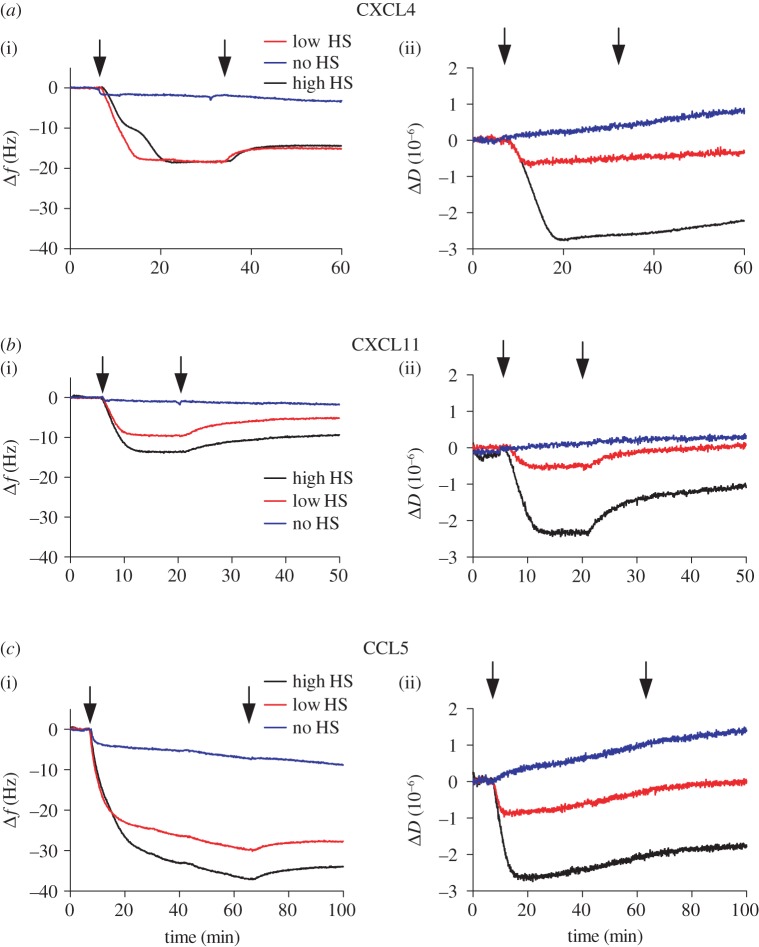
CXCL4-, CXCL11- and CCL5-mediated modification of high- and low-density HS films. (*a*) CXCL4, (*b*) CXCL11 or (*c*) CCL5 (500 nM) were passed over a QCM-D sensor displaying high-density (high HS) or low-density (low HS) HS films. Alternatively, chemokine was passed over a streptavidin-coated surface with no immobilized HS (no HS) to check non-specific binding (passivation). Subsequent changes in the (i) frequency (a decrease indicates bound chemokine) or (ii) dissipation (a decrease indicates HS film rigidification) as a function of time are plotted. Chemokine injection start and endpoints are indicated by arrows on each curve. The frequency plot for CCL5 demonstrates some non-specific binding (reduction in frequency) to the passivated surface (no HS) that is largely absent with the other chemokines used. This binding is associated with an increase in the dissipation on the HS-free surface, probably as a result of CCL5 polymerization, leading to a slight distortion of the dissipation measurement.

Figures 3 and 4 (black curves) demonstrate the changes in frequency and dissipation of a saturated HS surface after WT chemokines are flown over the surface. Several chemokines, representative of a broad range of oligomerization propensities, were chosen for this study in order to determine the effect of oligomerization on cross-linking HS: CCL7 (monomer [34]), CXCL11 (monomer at pH 4.5 [35]; weak dimer at pH 5.6 [36]), CXCL8 (dimer [52]), CCL2 (dimer [38]), CXCL4 (tetramer [41]) and CCL5 (polymer [43]). All chemokines produce a reduction in frequency, indicative of binding to the HS surface with the order of maximal signal change as follows: CCL5 (less than −30 Hz) > CXCL4 (−21 ± 3 Hz) > CCL2 (−20 ± 1 Hz) > CXCL11 (−14 ± 1 Hz) = CCL7 (−14 ± 1 Hz) > CXCL8 (−9 ± 1 Hz). These values indicate the level of accumulation reached at equilibrium by each chemokine on the HS surface with 500 nM chemokine in the solution phase, except for CCL5, which showed continued binding even after prolonged incubation for 60 min. Because the molecular weights of the chemokines are within 15% of each other, the values reflect to a first approximation the relative number of bound chemokine molecules, although their exact localization within the HS film, and effects on the morphology of the HS film may also affect the frequency shift. It is important to note that the maximal accumulation is not determined exclusively by the chemokine–HS affinity but rather affinity coupled with the propensity of the chemokine to oligomerize; thus it is not surprising that CCL5 and CXCL4 showed the greatest accumulation because they form polymers and tetramers, respectively, and also have the highest apparent affinity for HS of the chemokines tested [33]. These QCM-D experiments also demonstrate the variable rates of chemokine dissociation from the HS surface, where CXCL4, CXCL11 and CCL5 have slower rates of dissociation than CXCL8, CCL2 and CCL7, consistent with prior SPR kinetic data and the higher HS affinities of the former group [33]. CXCL8 also has a notably slow rate of association, again consistent with its relatively low affinity for HS [33].

In addition to providing insight into relative variations in the amount of chemokine bound to the HS films, QCM-D also enables simultaneous monitoring of the effect of chemokine binding on the rigidity of the HS surface through the dissipation readout, Δ*D* [50]. All chemokines produced a reduction in dissipation of the HS film indicating increased rigidity or reduced softness. However, the magnitude of the effect broadly distinguished two groups of chemokines. Chemokines that had a modest maximal effect on HS film rigidity (less than 2 dissipation units) include CXCL8 (−0.6 dissipation units), CCL7 (−1.2 dissipation units) and CCL2 (−1.3 dissipation units) (figure 3). The second group, which produced larger maximal reductions in dissipation (more than 2 dissipation units), and therefore greater HS film rigidification, includes CXCL4 (−2.8 dissipation units), CXCL11 (−2.4 dissipation units) and CCL5 (−2.7 dissipation units) (figure 4). After washing with buffer, dissociation of the chemokines from the HS film resulted in a reversal of HS film rigidification. In the cases of CXCL8, CCL2 and CCL7, the dissipation signal returned to pre-chemokine levels following buffer wash due to their relatively rapid rates of dissociation from the HS surface (figure 3). Conversely, the slower rates of chemokine dissociation observed for CXCL4, CXCL11 and CCL5 (figure 4) resulted in only small increases in dissipation following buffer wash. CXCL11 demonstrated an initial rapid loss of chemokine (indicated by the increase in frequency; figure 4*b*(i)), which then plateaued, and the dissipation signal showed a similar pattern (figure 4*b*(ii)). Thus, modification of the HS film softness is a direct result of chemokine binding, the magnitude of which is chemokine dependent. Moreover, the dissociation rates of different chemokines dictate the duration of the modifications, and result in both transient and more long-lasting effects.

### 2.2. Chemokine-mediated effects on heparan sulfate film rigidity are dependent on the surface density of the heparan sulfate chains

Given our previous observations of density-dependent chemokine–HS interactions by SPR [32,33], we investigated the density dependence of chemokine-mediated HS film rigidification using QCM-D. QCM-D sensor surfaces were functionalized as described above, but with only a third of the amount of HS (−7 Hz; ‘low-density HS surface’) immobilized relative to the saturated HS surface (−24 Hz; ‘high-density HS surface’) (figure 2) [51].

As shown in figures 3 and 4 (red curves), the low-density HS surface results in a reduction of the maximal level of bound chemokine, with the exception of CXCL4, with the most dramatic reductions observed for CXCL8, CCL2 and CCL7 compared with more moderate reductions with CXCL11 and CCL5. Conspicuously, CXCL8, CCL2 and CCL7 produced no reduction in dissipation in contrast with their effects on the high-density HS surface (figure 3), indicating that the rigidifying effect is reduced on the low-density HS surface. In the case of CCL2 and CCL7, the lack of measurable dissipation shift is despite their ability to bind to the surface, as indicated by the frequency measurements; in the case of CXCL8, very little chemokine even bound to the low-density HS surface (figure 3*a*). By contrast, CXCL4, CXCL11 and CCL5 were able to rigidify the low-density HS film substantially as indicated by the marked decrease in dissipation (figure 4). As with the high-density surface, CCL5 and CXCL4 produced a greater reduction in dissipation on the low-density surface than CXCL11. Although the effects were diminished in comparison with the high-density HS surface, the fact that these three chemokines could still substantially modify the low-density HS surface suggests that they can overcome the greater distance between HS chains and/or reduced density of HS binding sites to effectively rigidify the film.

### 2.3. Chemokine-mediated heparan sulfate rigidification is facilitated by oligomerization

**Figure 5.**
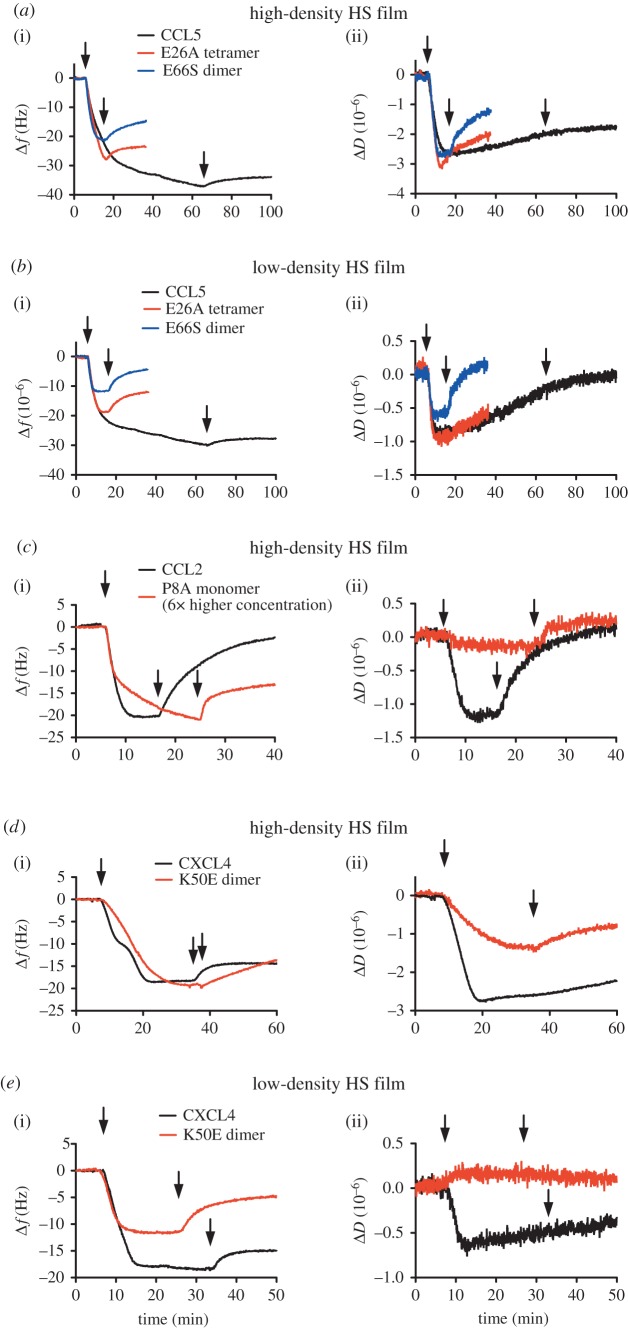
The role of chemokine oligomerization in modification of high- and low-density HS films. (*a,b*) CCL5 polymer or its oligomerization mutants, E26A tetramer or E66S dimer (500 nM), (*c*) CCL2 dimer (500 nM) or its oligomerization mutant P8A monomer (3 µM), (*d,e*) CXCL4 tetramer or its oligomerization mutant, K50E dimer (500 nM) were passed over high-density or low-density HS films and subsequent changes in the (i) frequency (a decrease indicates bound chemokine) and (ii) dissipation (a decrease indicates HS film rigidification) as a function of time, were monitored and plotted. Chemokine injection start and endpoints are indicated by arrows.

In our previous studies on the affinity and interaction kinetics of chemokines with GAGs by SPR, we observed a striking role of chemokine oligomerization through the use of oligomerization-deficient chemokine mutants [32,33]. The results motivated experiments to explore whether oligomerization plays a role in the observed chemokine-mediated rigidification of HS films using QCM-D. For this purpose, we used an E26A mutant of CCL5 (which forms tetramers) and an E66S mutant (which forms dimers) [53] for comparison with WT CCL5 (which forms polymers) [43]. Addition of WT CCL5 to a high-density HS surface produced a reduction of the dissipation measurement (−2.7 dissipation units) comparable with the E66S dimer (−2.7 dissipation units) but less than the E26A tetramer (−3 dissipation units) (figure 5*a*(ii)). This lower effect of WT CCL5 may be due to the effects of non-specifically bound protein, as described in the legend of figure 4. The QCM-D frequency measurement also demonstrated that the E66S dimer, and to a lesser extent the E26A tetramer, dissociated more rapidly from HS films than WT CCL5 following buffer wash (figure 5*a*(i)). On low-density HS surfaces, WT CCL5 and the E26A tetramer had the same maximal effect on dissipation (−1 dissipation unit) while the E66S dimer showed a reduced maximal effect (−0.6 dissipation units), presumably due to a reduced level of bound E66S dimer compared with WT CCL5 and the E26A tetramer (figure 5*b*). This small E66S-mediated reduction in dissipation was also entirely lost following buffer washing, in contrast with the E26A tetramer. Together these data suggest that although the dimeric form of CCL5 is able to rigidify the HS chains, higher-order oligomers are necessary for maximal binding and retention of CCL5 on the HS surface.

We also explored the role of oligomerization on CCL2-mediated HS rigidification using a P8A mutant that forms monomers instead of WT dimers [46]. Addition of the P8A monomer to the high-density HS surface resulted in very little binding at the same concentration as WT CCL2 (data not shown); thus a sixfold higher concentration of P8A was used to produce sufficient binding and enable comparison with the WT chemokine (figure 5*c*). Despite significant binding of P8A compared with WT CCL2, no reduction in dissipation was achieved. This finding suggests that oligomerization of CCL2 is necessary to produce high-affinity HS interactions and enable modification of HS chains, in agreement with the previous interpretation of SPR data [32].

Finally, we compared WT CXCL4 (tetramer) and a K50E mutant dimer to assess the effect of tetramer formation on HS film rigidification [54]. Previous characterization of this chemokine/mutant pair by SPR showed that dimeric K50E has a weaker affinity for HS primarily due to a more rapid off rate [33]. This finding was replicated here, as demonstrated by the frequency measurements; CXCL4 showed initial loss of bound protein followed by a levelling out, whereas less of the K50E dimer bound, and it was continuously released from the HS surface as buffer was flowed over (figure 5*d*). WT CXCL4 produced a significantly greater rigidifying effect on high-density HS films (−2.8 dissipation units) in comparison with the K50E dimer (−1.4 dissipation units). This finding was also replicated on low-density HS films, where K50E produced no effect on dissipation despite the significant levels of bound protein, in contrast to WT CXCL4 (figure 5*e*). These data suggest that the ability of CXCL4 to rigidify HS chains is dependent on its ability to form tetramers, which also promotes retention of the chemokine on the HS surface. Also of note is that a reproducibly observed discontinuity in the frequency change for binding of WT CXCL4 to the high-density HS surface is not replicated on the low-density surface or with the K50E mutant. The cause of the discontinuity is unknown but seems likely to be dependent on tetramer formation or a dimer–tetramer equilibrium, where the nature of the interaction of CXCL4 with HS changes at a given level of bound CXCL4.

### 2.4. Chemokine-mediated heparan sulfate cross-linking is a major cause of heparan sulfate film rigidification

**T**he QCM-D experiments described above demonstrate that certain chemokines can increase the rigidity of HS films and that this property is dependent on their ability to oligomerize. However, this assay does not directly address whether the observed rigidification is due to cross-linking of the HS chains (which has potential relevance to proteoglycan clustering, and related functional effects [29,55]), or whether the HS chains simply wrap around the chemokine. Importantly, the HS anchor points (e.g. biotin sites) are estimated to be 5 nm apart from each other on the high-density surfaces, whereas on the low-density surfaces they are approximately 9 nm apart [29,49,51]. In an earlier study, the chain length of the bound HS was also estimated to be 20 monosaccharides or approximately 10 nm [29]. Taken together, these dimensions suggest that chains from adjacent oligosaccharides should be able to contact each other, thereby allowing for cross-linking [29].

To address this question, we turned to FRAP to directly examine the lateral mobility of the HS chains in the presence and absence of chemokine. For these experiments, an HS film with laterally mobile HS anchor points was created by placing a lipid bilayer doped with biotinylated lipids onto a glass slide, followed by fluorescently labelled streptavidin and finally biotinylated HS (figure 2*b*) [29,49]. In this experimental set-up, a slowing of the fluorescence recovery reflects the reduced diffusion of streptavidin due to cross-linking of the attached HS chains.

**Figure 6.**
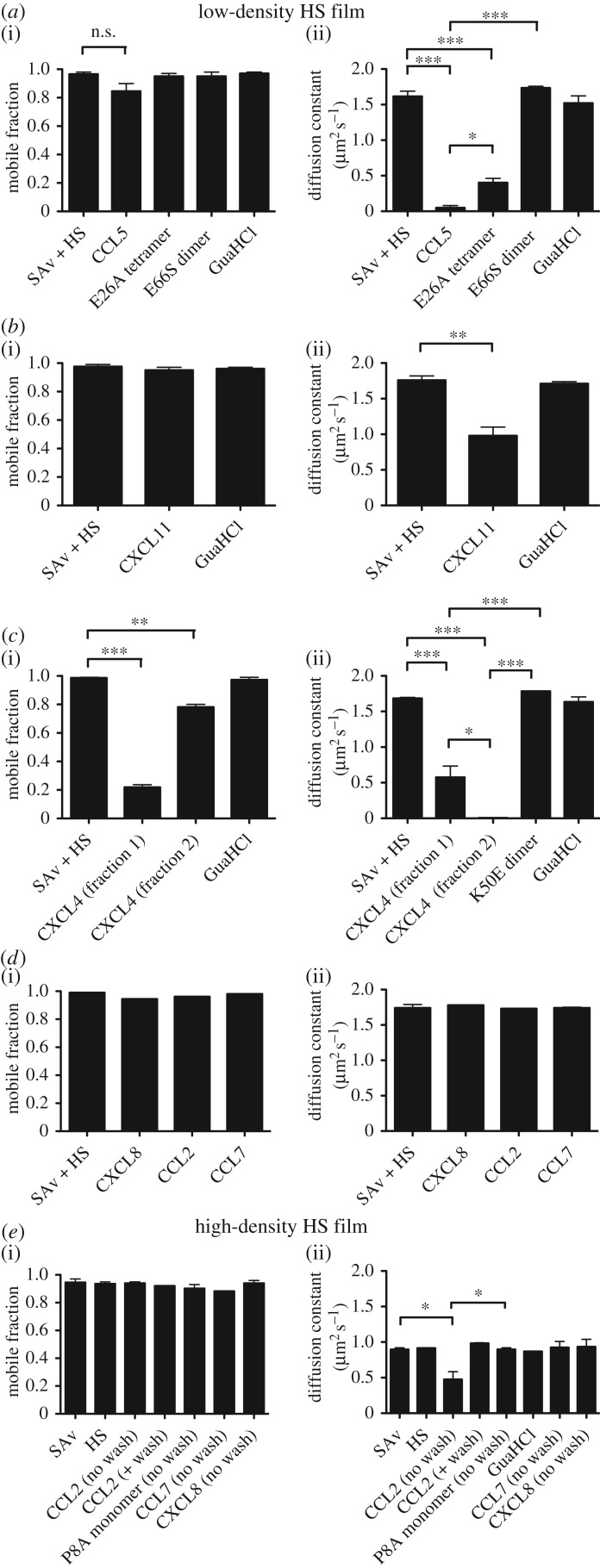
Chemokines cross-link HS chains dependent on oligomerization; quantitative FRAP analysis. (*a*) CCL5 polymer or its oligomerization mutants, E26A tetramer or E66S dimer (500 nM), (*b*) CXCL11 (500 nM), (*c*) CXCL4 tetramer or its oligomerization mutant, K50E dimer (500 nM), (*d*) CXCL8, CCL2 or CCL7 (500 nM) were incubated on lipid bilayers supporting fluorescently labelled streptavidin to which HS chains were attached to create low-density HS surfaces. (*e*) CCL2 dimer (500 nM), its oligomerization mutant P8A monomer (3 µM), CCL7 (500 nM) or CXCL8 (500 nM) were incubated on lipid bilayers supporting fluorescently labelled streptavidin to which HS chains were attached to create high-density HS surfaces. FRAP was monitored and data fitted to provide (i) the total mobile fraction and (ii) the associated diffusion constant; data are plotted as the mean ± s.e. of two independent experiments (*n* = 2). n.s., not significant, *p < 0.05, **p < 0.01, ***p < 0.001, as determined using a one-way ANOVA with post-test comparison.

**Figure 7.**
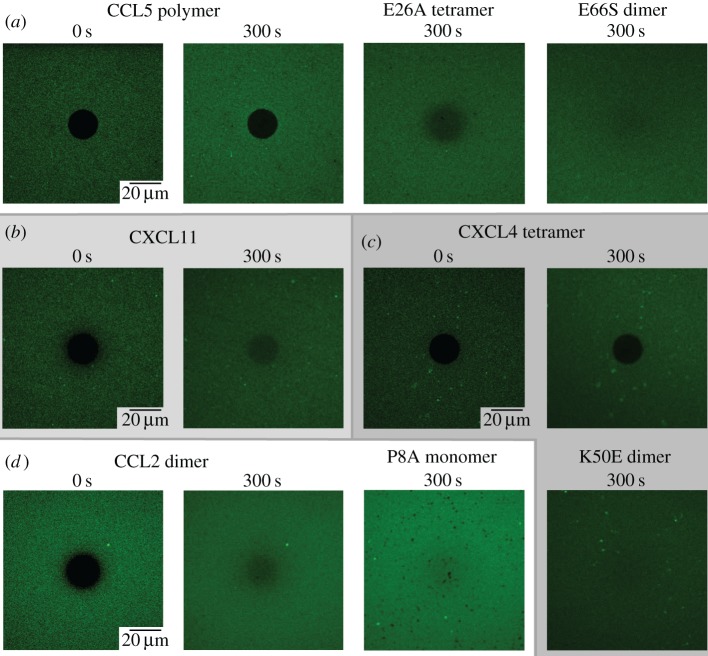
Chemokines cross-link HS chains dependent on oligomerization; representative FRAP micrographs. (*a*) CCL5 or its oligomerization mutants, E26A tetramer or E66S dimer (500 nM), (*b*) CXCL11 (500 nM), (*c*) CXCL4 or its oligomerization mutant, K50E dimer (500 nM) or (*d*) CCL2 (500 nM) or its oligomerization mutant, P8A monomer (3 µM) were incubated on a lipid bilayer supporting fluorescently labelled streptavidin to which HS chains were attached. Following bleaching and recovery analysis, images of the bleached spot were taken to provide visual assessment of the extent of maximal bleaching (0 s, images shown for WT chemokines only) and recovery from bleaching after 300 s (representative of two experiments).

In the absence of chemokine, a diffusion rate of 1.6 µm^2^ s^−1^ was measured for fluorescently labelled streptavidin. Upon addition of CCL5 followed by a wash step, it was reduced to 0.05 µm^2^ s^−1^ (figures 6*a* and 7*a*). By comparison, CXCL11 produced a lesser, but significant, reduction in diffusion to 1.0 µm^2^ s^−1^ (figures 6*b* and 7*b*). In the case of CXCL4, the default model with one mobile and one immobile fraction fitted the data poorly, indicating that a more complex situation was operative than that observed for CCL5 and CXCL11 (which may or may not be related to the discontinuity seen in the QCM-D frequency data). An extended model with two mobile fractions with distinct diffusion coefficients fit the data well and suggested that 78% of the film had a significantly reduced diffusion constant (0.005 µm^2^ s^−1^) and 22% had a more rapid (0.6 µm^2^ s^−1^) but still significantly reduced diffusion compared with the film in the absence of CXCL4 (figures 6*c* and 7*c*). In all cases, guanidinium hydrochloride (GuaHCl) was incubated on the HS film to remove bound chemokine and to demonstrate that this treatment returned films to their original laterally mobile state. Thus, it seems likely that CXCL4-, CXCL11- and CCL5-mediated rigidification of the HS films observed in the QCM-D experiments is due to cross-linking of the HS chains.

The HS density used in the above FRAP experiments is likely to be comparable with the low-density HS surface used for QCM-D [29]; thus it is not surprising that addition of CXCL8, CCL2 or CCL7 produced no reduction of the HS diffusion constant as detected by FRAP (figure 6*d*) because no reduction in dissipation was observed in the QCM-D experiments (figure 3). We therefore examined whether this was also the case on a higher-density surface, where QCM-D showed a decrease in dissipation and thus a higher degree of rigidification than on the low-density HS films (figure 3). As indicated in figure 6*e*, the high-density HS surface was characterized by a lower starting diffusion constant (0.9 µm^2^ s^−1^) compared with the lower density HS FRAP surface (1.6 µm^2^ s^−1^), probably because of the greater density of streptavidin and the associated HS chains. When CCL2 was incubated with this surface, followed by buffer washing, no effect on the mobile fraction or diffusion constant was produced, presumably due to loss of bound protein, as suggested by rapid dissociation of CCL2 in the QCM-D experiments. However, if the FRAP experiment was performed without buffer washing, then CCL2 significantly reduced the diffusion constant to 0.5 µm^2^ s^−1^ (figures 6*e* and 7*d*). This suggests that CCL2 transiently cross-links HS chains to produce the effects on rigidification seen by QCM-D. CCL7 and CXCL8 had no effect on either the mobile fraction or diffusion constant of these higher density HS surfaces (figure 6*e*), despite the presence of bound protein (figure 3).

### 2.5. Chemokine-mediated heparan sulfate cross-linking is dependent on oligomerization

In order to expand our understanding of the role of chemokine oligomerization in HS chain modification, we also examined oligomerization-deficient mutants by FRAP. The CCL5 E26A tetrameric mutant produced a significant reduction of the diffusion constant (0.4 µm^2^ s^−1^) compared with the HS chains alone (1.6 µm^2^ s^−1^), but the reduction was much less than that produced by WT CCL5 (0.05 µm^2^ s^−1^). No observable effect was produced by the CCL5 E66S dimer (figures 6*a* and 7*a*). Similarly, while WT CXCL4 produced two fractions with reduced diffusion constants, the K50E dimer had no effect on the mobility of the HS chains (figures 6*c* and 7*c*). Finally, in contrast to WT CCL2 on the high-density HS FRAP experiment, the monomeric CCL2 mutant, P8A, had no effect on HS chain mobility (figures 6*e* and 7*d*). As in the QCM-D assays (figure 5*c*), P8A was administered at sixfold higher concentration than WT CCL2 to reach comparable degrees of binding to the HS film. These results demonstrate that CCL5, CXCL4 and CCL2 need to form higher-order oligomeric structures in order to cross-link HS chains.

## 3. Discussion

### 3.1. Mechanisms of chemokine-induced remodelling of heparan sulfate

Cross-linking or clustering of HS proteoglycans is known to have functional effects that result in glycocalyx remodelling and changes in adhesion and barrier permeability to cells [10,12,56], and chemokines would seem to be prime candidates for initiating such a process. Prior studies suggestive of cross-linking [29,32,33] motivated the present study to broadly investigate this phenomenon with a panel of chemokines and with biophysical methods that directly assess GAG film rigidification and cross-linking, and to explore the role of chemokine oligomerization.

**Figure 8.**
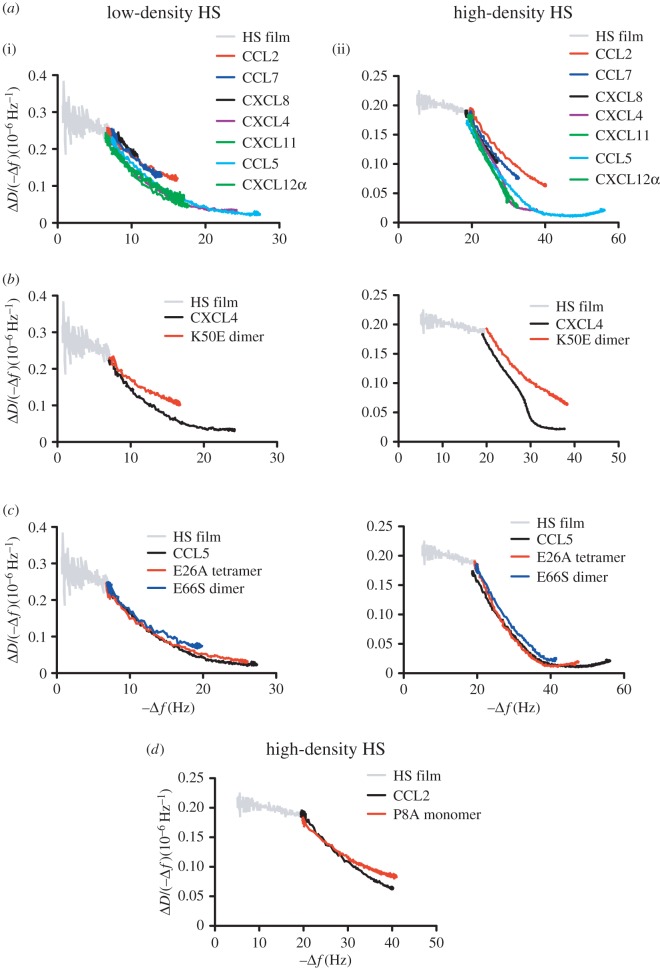
Chemokines and oligomerization mutants differentially modify HS film softness. In order to assess HS film modification as a function of bound chemokine, a measure of softness (Δ*D*/(−Δ*f*)) is plotted as a function of bound chemokine (−Δ*f*) on low- or high-density HS films for (*a*) WT chemokines, (*b*) CXCL4 or its oligomerization mutant, K50E dimer (500 nM), (*c*) CCL5 or its oligomerization mutants, E26A tetramer or E66S dimer (500 nM) or (*d*) CCL2 (500 nM) or its oligomerization mutant P8A monomer (3 µM). These curves were constructed using the same frequency and dissipation data plotted in figures 3–5. Data for CXCL12α were added in (*a*) for comparison, reproduced from fig. 4*b* in [29] but offset along the *x*-axis to adjust for the slightly higher HS surface densities used in that work.

The QCM-D experiments directly probed film rigidification, and the results demonstrate that all chemokines rigidify HS films, but to different extents, for different durations and probably by different structural mechanisms. The chemokines examined in this study have a broad range of affinities and oligomerization propensities; thus in order to compare rigidification at comparable levels of bound protein, a normalized representation of the data is useful (figure 8). In this representation, the ratio of dissipation over frequency shift (Δ*D*/−Δ*f*) for the HS film, bare or with chemokines, is a relative measure of film softness [50], and the negative frequency shift (−Δ*f*) is a relative measure of the amount of bound protein. As illustrated in figure 8*a*, the six WT chemokines studied here show only minor differences in their ability to rigidify the HS surfaces (i.e. decrease film softness) when compared at similar load levels (−Δ*f*), with the expected trend of CCL5, CXCL4 and CXCL11 showing greater rigidification than CCL2, CCL7 and CXCL8. For completeness, this plot also includes data for CXCL12α, which was investigated in a previous study [29]. The comparison indicates that CXCL12α is at least as potent as CCL5, CXCL4 and CXCL11 in rigidifying HS films at comparable protein load, a finding that is consistent with CXCL12α also being a very potent HS cross-linker [29]. From figure 8*a*, it is also clear that given sufficient levels of protein, all chemokines can rigidify the films to some degree. However, in general, the chemokines with the highest affinity for HS (CCL5, CXCL4 and CXCL11 [33]) showed the greatest propensity to rigidify the HS films on an absolute level (cf. minimal Δ*D*/−Δ*f* values reached in figure 8*a*) and also to remain stably associated with the HS films despite prolonged washing (cf. figures 3 and 4).

FRAP experiments were used to probe the lateral mobility of HS chains as an indicator of HS cross-linking. These results demonstrate that CCL5, CXCL4, CXCL11 and, to a lesser extent, CCL2 reduce the lateral mobility of HS chains, whereas CXCL8 and CCL7 have no effect at all. Clearly, HS cross-linking would be expected to contribute to HS film rigidification, but the distinct trends in the normalized representation of the QCM-D data (figure 8) and the FRAP data (figure 6) also imply that additional mechanisms of film rigidification must be at play. Possible mechanisms include HS chains individually wrapping around chemokines, or simply the crowding of the HS film by chemokines.

The ability of CXCL4 and CCL5 to oligomerize is clearly important not only for their affinity and slow off rates from GAGs, but also for their ability to rigidify and cross-link HS films. This was demonstrated with dimeric and tetrameric mutants of polymeric WT CCL5, both of which showed a reduced capacity to rigidify and remain stably associated with HS films (figure 5*a,b*); moreover, the E26A tetramer showed a reduced ability to cross-link the films relative to WT, while the E66S dimer showed no cross-linking under the conditions tested (figure 6*a*). Similarly, the CXCL4 K50E dimeric mutant showed a reduced capacity to bind, to remain associated with and to rigidify HS films compared with WT tetrameric CXCL4 (figure 5*d,e*), and it also lost the ability to cross-link HS (figure 6*c*). CCL5 and CXCL4 have relatively extensive GAG-binding epitopes in the context of their polymeric [43] (figure 1*f*) and tetrameric [41] (figure 1*e*) structures, respectively, which would facilitate their affinity for, retention on and ability to cross-link HS chains.

In the case of CXCL4, normalized softness plots also suggest that the WT chemokine is more effective in rigidifying HS films than the oligomerization-impaired mutant, even at equivalent levels of bound protein (figure 8*b*). It is notable that this is not so for CCL5, as the curves for the WT and mutant forms overlap closely (figure 8*c*). The comparable capacity of WT and mutant CCL5 to rigidify HS films when considered on a per molecule basis contrasts with their differential abilities to slow down HS diffusion (cf. figures 6 and 7). A possible explanation is that dimers, tetramers and polymers of CCL5 can all cross-link HS chains, but that the stability of the cross-links increases with oligomer size. In this scenario, the two HS binding patches per CCL5 dimer (figure 1*f*) bind two distinct HS chains, thus promoting film rigidification, but such cross-links are too short-lived to slow HS diffusion appreciably; larger oligomers enhance binding to the individual HS chains, and thus stabilize the cross-link and effectively slow HS diffusion. The example of CCL5 thus illustrates that chemokine oligomerization can differentially modulate the rigidification of HS matrices and the mobility of HS chains.

CXCL11 was also able to rigidify and cross-link HS films, although not as robustly as CXCL4 and CCL5 (figures 4 and 6*a–c*). The CXCL11 structure was solved as a monomer by nuclear magnetic resonance (NMR) but under conditions of low pH (pH 4.5), which generally destabilizes or dissociates chemokine into monomers or smaller oligomers [35]. At pH 5.6, however, NMR diffusion experiments indicate that CXCL11 forms weak dimers [36]. Dimers are also consistent with the level of accumulation of CXCL11 on heparin and HS as observed by SPR data, when compared with other chemokines of known oligomerization states and affinities [33]; thus we predict that dimerization is important for its HS modifying capacity, which would make for a rather extended GAG-binding surface (figure 1*b*). Several studies have also shown that chemokines oligomerize on GAGs and that GAGs stabilize chemokine oligomers, implying that chemokine oligomerization and GAG binding are thermodynamically coupled. Among the chemokines studied here, CXCL11 may represent a typical chemokine whose oligomerization is weak in solution but strongly promoted upon binding to HS.

CXCL8 and CCL2 form stable dimers in solution (figure 1*c,d*) [39,57]; however, because of their reduced affinity for GAGs [32,33], they are less efficient in modifying HS compared with CCL5, CXCL4 and CXCL11. CXCL8 has the lowest affinity for HS of all the chemokines examined in this study [33] and it showed no ability to cross-link HS chains (figure 6*d,e*). CCL2 did cross-link HS, although only transiently and only on high-density HS films (figure 6*d,e*). Similar to CCL5 and CXCL4, CCL2-mediated rigidification and cross-linking was dependent on oligomerization as monomeric P8A [46] was ineffective, even at a sixfold higher concentration than that used for the other chemokines (figures 5*c* and 6*e*). It was also inefficient in rigidifying high-density films, and normalized softness plots indicated that WT CCL2 was more effective than P8A in rigidifying the films on a per molecule basis (figure 8*d*). The fact that CCL2 requires oligomerization to rigidify and cross-link HS films makes intuitive sense given the position of its single GAG-binding epitope at opposite ends of the dimer (figure 1*c*).

CCL7 is a monomeric chemokine [34], and thus the fact that it did not cross-link HS (figure 6*d,e*) was unsurprising. However, it was much more effective in rigidifying the HS surfaces than the highly homologous CCL2 monomer P8A [46] (figures 3*c* and 5*c*). The inability of CCL7 to cross-link may be due to an inability to span epitopes between adjacent HS chains coupled with a slightly reduced affinity for HS compared with WT CCL2 [32]. The ability of CCL7 to rigidify HS films compared with P8A is consistent with its slightly higher affinity for HS than P8A, although even on a normalized per molecule basis, CCL7 has a greater ability to rigidify HS (figure 8*a,d*). As suggested previously [32], this is probably because CCL7 has a more dense and extended GAG-binding surface in the context of its tertiary structure compared with a single subunit of CCL2 (figure 1*a,c*). As it does not cross-link HS chains according to the FRAP data, its ability to rigidify HS may reflect condensation or wrapping of individual HS chains around a single CCL7 subunit.

Taken together with information on the presence and distribution of the GAG-binding epitopes on these chemokines (figure 1), the results suggest that oligomerization enhances the ability of chemokines to modify HS films by a number of inter-related factors: (i) enhanced affinity and accumulation on HS due to the simultaneous binding of multiple chemokine epitopes to HS; (ii) the ability to bridge gaps between GAG chains by producing structures with multiple, spatially separated GAG-binding sites such that the oligomer can bind to multiple HS chains at once; and (iii) the ability of multiple GAG-binding epitopes in the context of oligomers to promote chemokine rebinding. Not surprisingly, the length of the HS chain also influences cross-linking, with longer chains promoting higher affinity interactions [58] and greater rigidification than short-chain GAGs (e.g. dp6), as previously demonstrated for CXCL12α [29]. Furthermore, it seems likely that HS overall sulfation and fine structure will also play key roles in promoting cross-linking given their importance in chemokine–GAG interactions [33]. The results also suggest that the magnitude and duration of HS rigidification and cross-linking is specific to individual chemokines, reflecting fine tuning of individual chemokine functions; this adds more evidence to the idea that chemokine function may not be as redundant as initially surmised based on the apparent promiscuity of receptor–chemokine interactions [59].

### 3.2. Functional consequences of heparan sulfate rigidification and cross-linking

**Figure 9.**
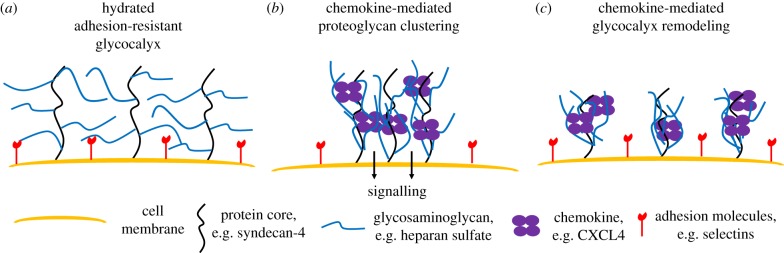
Chemokine-mediated GAG cross-linking may enable syndecan clustering and manipulation of glycocalyx structure to enable leukocyte migration events. (*a*) In the absence of chemokine, proteoglycans form a hydrated adhesion-resistant surface on the endothelium to control leukocyte movement. (*b*) Chemokine-mediated HS cross-linking may produce syndecan clustering, enabling subsequent signalling events. (*c*) Chemokine-mediated HS cross-linking may restructure the glycocalyx exposing endothelial adhesion molecules to circulating leukocytes. These potential chemokine functions could help describe the fundamental importance of chemokine–GAG interactions.

Chemokine–GAG interactions have been hypothesized to provide a mechanism for chemokine localization and formation of gradients that guide migrating cells to inflammatory sites [27,28]. Our data certainly support the idea that these interactions promote the retention and slow release of high affinity HS-binding chemokines that in turn may contribute to gradient formation. However, it is also possible that chemokine–GAG interactions are involved in structural modification of the endothelial glycocalyx, and possibly proteoglycan-dependent signalling (figure 9). This would be consistent with an emerging view that inflammatory cytokines remodel the ECM and endothelial cell glycocalyx to mediate leukocyte adhesion, migration and endothelial barrier permeability [10,12,29,56].

The endothelial glycocalyx, which is rich in HS-containing proteoglycans, has been described as a thick adhesion-resistant barrier to cell migration by imposing a physical layer, estimated as being 0.2–2 µm thick [6–8]. This depth has been suggested to inhibit accessibility to key molecules (e.g. selectins, integrins) involved in adhesion of leukocytes to the endothelium, an important step in transmigration [5]. Indeed a theoretical study suggested that the combination of intact glycocalyx structure and blood flow render it impervious to penetration by leukocyte microvilli [60]. Thus, modification of this structure would be necessary for initial adhesion events and subsequent transmigration. Tumour necrosis factor (TNF) stimulation of the endothelium has been shown to reduce the glycocalyx thickness and lead to enhanced cell adhesion due to the breakdown of this physical barrier [56]. Conversely, therapeutic protection of the glycocalyx structure reduces leukocyte adhesion following ischaemia/reperfusion [61] and has been described as a potentially wide-ranging therapeutic approach [11]. Mechanisms for TNF-mediated glycocalyx remodelling and permeability were proposed to involve production of proteases and proteoglycan shedding [10,12,56] and this has been shown to be the case for TNF-α-induced disruption of the glomerular endothelial glycocalyx [62], a process that can be inhibited by hydrocortisone and antithrombin [63].

Chemokines may be able to modulate the glycocalyx in similar ways. Chemokine cross-linking of HS chains, described here *in vitro*, may produce a reduced thickness and/or permeability of the glycocalyx layer *in vivo*. This would represent a novel function of chemokines where, prior to their established function in G protein-coupled receptor (GPCR) signalling and activation of leukocytes, they physically reorganize specific sites on the endothelial surface to mediate localized adhesion events. Importantly, CXCL12 and CCL5 have been reported to promote clustering of the proteoglycans syndecan-1 and -4 on HeLa cells [64,65], which may be a consequence of their ability to cross-link GAG chains as shown here. Syndecan-4 is a particularly logical player in the function of chemokines as one of its well-studied functions is in the formation of focal adhesions, which are important for cell migration [66]. The function of syndecan-4 has been associated with its ability to form clusters [55,67], where multiple HS side chains are necessary for cell adhesion unless rescued by antibody-induced clustering [68]. Furthermore, antibody-mediated syndecan-4 clustering has been shown to induce migration of endothelial cells [69]. These data suggest that HS cross-linking and subsequent clustering by ligands could be important in the behaviour of this proteoglycan in the context of chemokines as well. CCL5 and CXCL12 have also been shown to accelerate shedding of syndecans from the surface of cells [70,71], which again may be linked to HS cross-linking and proteoglycan clustering. In the case of CXCL12, the process was shown to be dependent on proteoglycans, but not on the CXCL12 cell surface receptor, CXCR4 [70], whereas in the case of CCL5, it was dependent on both syndecans and the receptor CCR5 [71].

In addition to affecting the physical structure of the glycocalyx, receptor-independent signalling of chemokines through proteoglycans has been observed for CXCL12 and CCL5 [65,70,72,73], and indeed, ligand-mediated clustering of syndecans appears to be a key mechanism for proteoglycan signalling [67]. For example, CCL5-mediated signalling through the mitogen-activated protein kinase pathway is dependent on the presence of GAGs and also upon its ability to oligomerize [72], reflecting a potential role for HS cross-linking. More generally, clustering of syndecan-4 serves to concentrate the proteoglycan into micro-domains that recruit and scaffold signalling molecules on the inside of the cell [66], and the ability of chemokines to oligomerize could be relevant in promoting this function. As HS chain density also affects binding of chemokines on the outside of the cell, the interaction between chemokines and HS may serve to synergistically concentrate chemokines and other cytokines on the cell surface. Overall, there are many reasons to expect that chemokine–GAG interactions play a more elaborate role in the overall process of cell migration than simply serving as the stationary beacons for migrating cells **(**figure 9).

In conclusion, here we extend a previous observation that CXCL12 can modify HS films to include other chemokines separated into a lower modifying group (CXCL8, CCL2 and CCL7) and a higher modifying group (CXCL4, CXCL11 and CCL5). The relative potency is linked to the ability of the chemokines to oligomerize coupled with their affinity for HS. The biological function of this behaviour remains to be defined; however, it may enable chemokines to cluster proteoglycans with concomitant effects on proteoglycan signalling. Furthermore, remodelling of the glycocalyx by cross-linking GAGs may represent an additional function related to the importance of chemokine–GAG interactions during leukocyte migration *in vivo*.

## 4. Experimental procedures

### 4.1. Materials

Chemokines and mutants were recombinantly expressed and purified as described previously [32,74,75]. Streptavidin and fluorescently labelled streptavidin (Sigma Aldrich), dioleoylphosphatidylcholine (DOPC), dioleoylphosphatidylethanolamine-CAP-biotin (DOPE-CAP-biotin) (Avanti Polar Lipids, Alabaster, AL, USA) were purchased. HS from porcine intestinal mucosa was kindly provided by H. Lortat-Jacob (Institut de Biologie Structurale, Université Grenoble Alpes, Grenoble, France) and biotinylated as described previously [51]. The HS was found to have an average molecular weight of 12 kDa, a polydispersity of 1.6 and an average of 1.4 sulfates per disaccharide [76].

### 4.2. Quartz crystal microbalance with dissipation monitoring

QCM-D experiments were performed using a Q-Sense E4 system (Biolin Scientific, Västra Frölunda, Sweden) as previously described in detail [29,49]. Briefly, gold-coated sensors (QSX301; Biolin Scientific) were treated in an UV/ozone chamber for 30 min and then coated overnight by immersion in OEG disulfide and biotinylated OEG thiol (1000 : 1 molar thiol equivalents, 1 mM total concentration) dissolved in ethanol. Sensors were then rinsed with ethanol to remove any residual reagent and mounted into QCM-D Flow Modules (Biolin Scientific) before equilibration in running buffer (10 mM HEPES, 150 mM NaCl, pH 7.4).

Streptavidin was then grafted onto the sensor surface by being passed over in running buffer, first at 1 µg ml^−1^ to confirm no depletion from bulk flow (i.e. due to binding to the walls of the fluidic system), and then at 20 µg ml^−1^ until saturation was reached (typically −24 Hz). Subsequently, immobilization of biotinylated HS was performed by flowing a 2 µg ml^−1^ or 5 µg ml^−1^ suspension in running buffer over the sensor surface until a lower HS density (−7 ± 1 Hz; ‘low-density HS surface’) or until saturation (−24 ± 1 Hz; ‘high-density HS surface’) was reached, respectively. Chemokine or chemokine mutants (typically 500 nM) were then passed over these surfaces in running buffer and the frequency and dissipation changes monitored. This concentration was chosen in order to elicit modifying effects on this biomimetic HS film. It is hard to compare this to relevant chemokine concentrations *in vivo* as the effect of ECM interactions is likely to generate a high, and currently non-defined, localized chemokine concentration within the glycocalyx and tissues. Surfaces were regenerated by removing bound chemokine (a maximum of three times) using 2 M GuaHCl in ultrapure water which returned the frequency and dissipation to pre-chemokine levels and had no detrimental effect on subsequent chemokine binding (as demonstrated previously [49]). Data are presented as normalized shifts in the resonance frequency, Δ*f* = Δ*f_n_*/*n* (*n* being the overtone number), and shifts in the dissipation, Δ*D*, obtained from the fifth overtone of the QCM-D sensor in each instance. Any other tone (*n* = 3, 5, … , 13) would have provided similar results. Measurements were repeated twice and data displayed are representative of these independent experiments.

### 4.3. Fluorescence recovery after photobleaching

FRAP experiments were undertaken as described previously [29]. Small unilamellar vesicles (SUVs, 100 µg ml^−1^ total lipid) were prepared by sonication [77] in FRAP buffer (10 mM Hepes, 150 mM NaCl, pH 7.4) supplemented with 2 mM CaCl_2_. SUVs containing 99.5 mol% DOPC and 0.5 mol% DOPE-CAP-biotin (for low-density HS surfaces), or 95 mol% DOPC and 5 mol% DOPE-CAP-biotin (for high-density HS surfaces) were incubated (30 min, room temperature) in wells formed on a pre-conditioned glass slide to create a biotinylated supported lipid bilayer *in situ*. Fluorescently labelled streptavidin (10 µg ml^−1^ in FRAP buffer) was then grafted onto the surface by incubating for 20 min at room temperature, followed by washing (two times with FRAP buffer). Biotinylated HS (10 µg ml^−1^ in FRAP buffer) was subsequently added and incubated for 30 min at room temperature. Chemokine or chemokine mutants were then incubated (typically 500 nM in FRAP buffer) with the HS film for 20 min at room temperature prior to the FRAP experiments. GuaHCl in ultrapure water was incubated on the HS film to remove bound chemokine and demonstrate that this returned films to their original laterally mobile state. FRAP experiments were performed using a confocal microscope (LSM 510; Zeiss, Oberkochen, Germany), with or without washing of the HS film with FRAP buffer. For recovery analysis, a series of images of the bleached spot were taken during the recovery period (approx. 5 min). The images were then analysed using ‘time-resolved profile analysis', a custom-made Matlab (MathWorks, MA, USA) protocol [29,78]. By default, a lateral diffusion model with one mobile fraction and one immobile fraction was used and found to fit most of the data well. Where this was not the case, an extended model was used featuring two mobile fractions, each with a distinct diffusion constant, and no immobile fraction.
